# How Hospital Tours Affect Preoperative Anxiety in Mothers with Children Undergoing Open-Heart Surgery in Iran: A Quasi-Experimental Study

**DOI:** 10.30476/ijcbnm.2020.82761.1084

**Published:** 2020-07

**Authors:** Asal Amintojari, Manijeh Nourian, Lida Nikfarid, Parasto Ojian, Malihe Nasiri

**Affiliations:** 1 Department of Pediatric Nursing, School of Nursing and Midwifery, Shahid Beheshti University of Medical Sciences, Tehran, Iran; 2 Department of Biostatistics, School of Nursing and midwifery, Shahid Beheshti University of Medical Sciences, Tehran, Iran

**Keywords:** Anxiety, Congenital heart disease, Orientation, Parents, Preoperative period

## Abstract

**Background:**

Parents tend to experience considerable amounts of anxiety before their children undergo open heart surgery. This study was conducted to assess the effects of taking a hospital tour on preoperative anxiety in the mothers of children undergoing open heart surgery.

**Methods:**

In this quasi-experimental study, 96 mothers from Shahid Modarres Hospital in Tehran, Iran, during April to December 2018, were selected through convenience sampling and were assigned to three groups using simple randomization. The oral instruction group (N=32) attended two oral instruction sessions; the hospital tour group (N=32) participated in tours of the operation room and intensive care unit; the control group (N=32) was prepared according to the ward’s routine. Preoperative anxiety was evaluated using the Amsterdam Preoperative Anxiety and Information Scale and the State Trait Anxiety Inventory. Data were analyzed in SPSS-20. The ANOVA, paired t-test and Tukey’s test were used for the data analysis. The level of statistical significance was set at P<0.05.

**Results:**

The mothers’ anxiety about surgery (F=30.99, P≤0.001) and their scores of state anxiety (F=6.02, P<0.001) differed significantly among the three groups after the intervention. A significant difference was observed between the oral instruction and control groups (P<0.001) and the hospital tour and control groups (P<0.001) regarding the surgery-related anxiety scores. A significant difference was also observed between the oral instruction and control groups (P=0.002) regarding the mothers’ state anxiety scores.

**Conclusions:**

The results suggest the greater efficiency of oral instructions versus hospital tours. Nurses can use oral instructions for reducing surgery-related anxiety and state anxiety of mothers before their toddlers’ open heart surgery.

**Trial Registration Number:** IRCT20180904040944N1.

## INTRODUCTION

Congenital Heart Disease (CHD) is a defect in the function and structure of the heart that results in prolonged and frequent hospitalizations and multiple surgeries. ^1^ About 9.1 children were born with CHD per 1000 live births, and global prevalence of CHD continues to increase. In Asia, the prevalence is high, at about 12.3 for every 1000 live births. ^2^

The hospitalization of children in Intensive Care Units (ICUs) for undergoing open heart surgery is associated with high levels of stress and anxiety in the parents. ^3 , 4^ Results of a study showed that 51% of the parents of children undergoing surgery had probable anxiety. ^5^ Preoperative anxiety is more common in mothers compared with fathers. ^6^ Mothers of children who had undergone cardiac surgery experience symptoms of acute stress and a sense of inability, confusion, and panic. ^7^

Parents’ anxiety is usually caused by factors such as operation complexities, unknown outcomes, fear of medical equipment, observing their anesthetized or sedated child, ^8 , 9^ lack of awareness, uncertainty and fear of the child’s future and life. ^10^ Parents’ anxiety may cause behavioral and emotional problems and decrease their ability to manage and take care of their child, reduce their quality of life ^11 , 12^ and elongate the child’s post-operation recovery. ^13^ The transmission of parents’ anxiety may intensify the child’s anxiety as well, and the child may then demonstrate symptoms of psychosis, postoperative maladaptive behavior, nightly fears, separation anxiety, nutritional disorders, and enuresis. ^14^ Additionally, parents’ anxiety may negatively affect the children’s perioperative pain sensations ^6^ and increase pulmonary complications such as pneumonia and atelectasis and elevate the child’s heart rate and peripheral vascular resistance. ^14^ Moreover, it may disturb the child’s ability to eat food and sleep and damage the child’s physical, mental and social status, which may interfere with treatment side-effects and complicate the disease management. ^15^

Before an operation, the parents should be informed and prepared by the health care team to act as an active member in their child’s treatment. ^3^ Emphasis on family-centered care and provision of information in an appropriate way is, therefore, crucial for reducing the preoperative anxiety of the parents, especially the mothers. ^6^ The preoperative preparation of the parents, which is an important professional responsibility of the nurses, ^16^ may be performed through different methods. ^14^ Different efficiencies have been reported for different methods of preoperative preparation of the parents; ^17 , 18^ however the best method is not yet clear. Choosing the best method for this purpose helps reduce the parents’ anxiety and increase their ability to understand information about their child’s condition. Preoperative preparation with oral instructions is one of the most common methods of conveying information to the parents that has various benefits. ^19^ Nonetheless, even if the information provided is satisfactory and the mothers find it comprehensible, only half of the presented information will stick in their mind, ^17^ which may reduce the effectiveness of this preparation method. Moreover, evidence also shows that taking hospital tours can reduce anxiety through the promotion of information, knowledge and experience and the improvement of cognitive and verbal power ^20 , 21^ though some studies have failed to confirm the effectiveness of this method. ^22 , 23^

In Iran, most studies show the effect of hospital tour on preoperative anxiety of the patients themselves. ^18 , 20 , 24^ In one study, mother’s anxiety was examined in children who had undergone general surgery, and the results showed the positive effect of individual and group tours on the children and mothers. ^19^ Researchers did not find a study conducted on the effect of this method on anxiety in mothers of children who had undergone open-heart surgery. According to the results of previous studies and the existing challenges of choosing the best method for the preoperative preparation of parents before their child’s open heart surgery and contradictory results of hospital tour, the present study aimed to examine the effect of hospital tours on the preoperative anxiety of the mothers of children with CHD and compare it with the routine and oral instructions methods.

## MATERIALS AND METHODS

**Figure 1:**
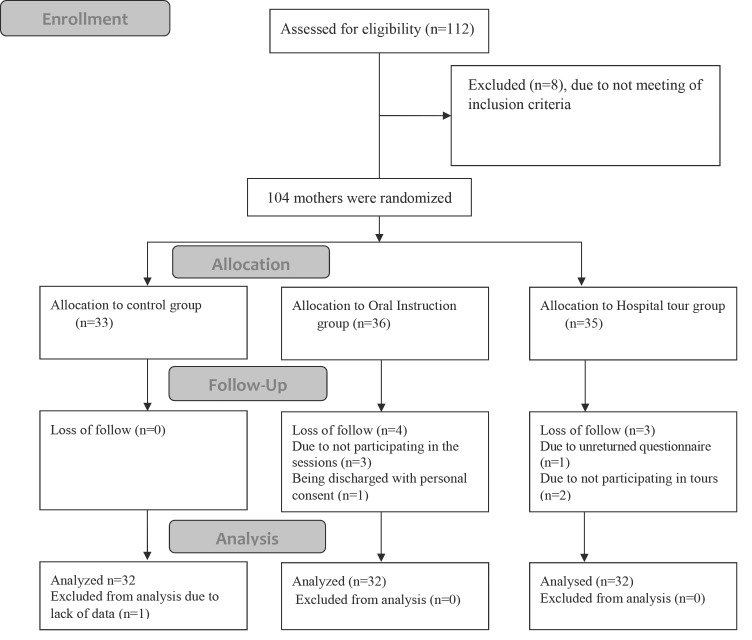
Consort flow diagram of participants

This quasi-experimental study was conducted with a pretest-posttest design and a control group. The study population consisted of the mothers of toddlers with congenital heart disease who had hospitalized their children for open heart surgery at Modarres hospital in Tehran, Iran, which has a pediatric cardiology ward for pre- and post-operative care, and ICU of pediatric cardiac surgery with 8 beds. This study lasted from April 19 to December 16, 2018. The mothers were selected through convenience sampling and randomly assigned to three groups, including the oral instruction group, the hospital tour group and the control group (receiving the routine care provided by the ward), using simple randomization. For this purpose, at the beginning of each three-week period, by simple drawing, the researchers decided about the groups the participants would be assigned to. To prevent communication between the participants, we performed sampling with a one-week interval for each group. Sampling was continuous; that is, it continued until the required sample size was achieved (Figure 1). Considering a type-I error of 5% and a power of 95%, we calculated the sample size as 32 per group. Given that the study had three groups (r=3), an ANOVA table was used to calculate the sample size. ^25^ Using this table requires specifying the amount of Δ/σ; since σ (standard deviation) was 3.4 and Δ equaled the difference between the maximum (6.5) and minimum (3.1) mean score of anxiety, Δ/σ was calculated as 1. ^20^

The study inclusion criteria consisted of having no other underlying or chronic diseases in the child, not having psychological diseases or anxiety disorders in the mother, the mother being 20-50 years old, being the child’s biologic mother, not having taken any anxiolytics at least 24 hours before the operation, and being able to speak Persian. The mothers who did not participate in one of the oral instruction sessions or one session of the hospital tour, were not willing to continue their participation, whose child’s operation was canceled, whose child had experienced alterations in his/her hemodynamic and physical conditions the day before the operation, and whose child had required an emergency operation or had died were excluded from the study.

The data collection tools included a demographic questionnaire for the mother (educational level, number of children, age) and child (age, sex, disease, length and history of hospitalization), the Amsterdam Preoperative Anxiety and Information Scale (APAIS) to measure the preoperative anxiety of the mothers in relation to their child’s operation, and the State Trait Anxiety Inventory (STAI) to measure the mothers’ state anxiety.

The APAIS is a self-report and short questionnaire designed by Dutch group of Moermann in 1996. They report two dimensions, which explain 72% of the variance. Cronbach’s alpha for the four anxiety items (items1, 2, 4, 5) was 0.86 and for the information requirement (items 3 and 6) was 0.68. The correlation between both dimensions was 0.31. The anxiety items of APAIS correlates with the STAI with r=0.74. ^26^ Reliability and validity of APAIS also have been reported to be acceptable in various studies ^27 , 28^ and it has been used to investigate parental anxiety before surgery. Scoring is based on a 5-point Likert scale from ‘Completely Disagree’ (1 point) to ‘Completely Agree’ (5 points). The score of the first dimension ranges from 4 to 20 and that of the second dimension from 2 to 10. In this tool, higher scores indicate a higher preoperative anxiety and greater need for preoperative information. ^26^ The scale was evaluated by Nikandish et al. (2007) in Iran. Construct validity was assessed by exploratory factor analysis and showed two dimensions which explained 72% of the total variance. Cronbach’s alpha for the anxiety dimension and for information requirement dimension was 0.84 and 0.82, respectively. The correlation between the anxiety dimension and the STAI-State was 0.68, and that between the information dimension and the STAI-State was 0.50. ^29^

The STAI includes a separate self-report scale to measure state and trait anxiety; it was designed in 1970 by Spielberger. Internal consistency coefficients for the scale have ranged from 0.86 to 0.95; test-retest reliability coefficients have ranged from 0.65 to 0.75. ^30^ The STAI is frequently used in Iranian population. In determining the psychometric properties of it in Iranian society, Mahram (2007) reported that the reliability of the scale for the norm and criterion groups was α=0.94, and the mean anxiety in the criterion group was significantly higher than the norm group (P<0.01). ^31^

This study used the state anxiety section of this inventory, which consists of 20 statements that measure the subject’s anxiety at the moment of responding to the questionnaire. The scores of this questionnaire range from 20 to 80. Higher scores indicate a higher anxiety level. This tool has been used in different studies in diverse societies and is reported to have satisfactory reliability and validity. ^6 , 24^

To determine the qualitative content validity of the obtained data, the noted questionnaires were reviewed by 14 experts and faculty members of the Nursing and Midwifery Faculty of Shahid Beheshti University of Medical Sciences and experts and nurses at Shahid Modarres Hospital in Tehran. Additionally, to determine the reliability of the internal consistency, Cronbach alpha was calculated for the STAI (α=0.88) and APAIS (α=0.78). Moreover, the questionnaires were distributed among 15 eligible mothers so as to define its face validity by examining their understanding of the statements and making the necessary modifications.

The parents’ preparation started in the morning of the day before their child’s operation (i.e. 24 hours before the operation), which is considered the right time for preoperative preparation. ^32^ Informed consent was taken from them after explaining the study design and objectives. The level of operation-related anxiety, state anxiety, need for information and demographic information were measured/ in all the three groups. All the questionnaires were completed by the mothers in the presence of the researcher. In the oral instruction group, the mothers separately took part in two instructional sessions (one held in the morning and another in the afternoon at the pediatric cardiology ward’s classroom) followed by a collective 100-120-min session. The morning session covered oral instructions about the disease, surgery and its possible complications, anesthesia procedure for the child, pre- and post-operative care measures and the child’s appearance when leaving the operation room; images were demonstrated in this session. Then, between 4 and 6 PM, the mothers were briefed on how their child was being monitored; the reason for using a ventilator; the child’s nourishment during mechanical ventilation; the onset of oral nourishment; devices such as arterial line, urinary catheter, chest tube, nasogastric tube; and their care and the time of transmission to the ward. Finally, at the end of the session, necessary explanations were provided for mothers based on their answers to items 3 and 6 in the APAIS.

In the hospital tour group, the mothers separately participated in two 60-min hospital tours in the morning and afternoon. They were first informed about the principles of entering the operation room and ICU and were then taken to the operation room in the morning tour and the ICU in the afternoon tour; the same information given in the oral instruction sessions was presented to them as well. At the end of the hospital tour, the necessary information was presented to them based on their answers to the APAIS. In both intervention groups, all the explanations were provided in simple words without using specialized terms, and the mothers were able to ask their questions without any limitations. The information provided was approved by experts in pediatric open heart surgery, and the mothers also received conventional preparation care in both groups.

In the conventional preparation group, the mothers received instructions according to the routine care plan of the ward. They were not instructed by the researcher and were merely given information on the NPO time, preparation of the surgery site, and the child’s is transfer to the ICU after the operation through a 5-min oral instruction session.

In all the three groups, the mothers’ operation-related and state anxiety and need for information were evaluated one hour before the child was transferred to the operation room. To comply with the ethical rules, the control group received oral explanations after the study was over and their questions were answered. 

The ANOVA, paired t-test and Tukey’s test were used to analyze the data, which was performed in SPSS-20. The level of statistical significance was set at P<0.05. This study was approved at Shahid Beheshti University of Medical Sciences under the ethical code of IR.SBMU.PHNM.1395.683. All mothers were informed of the objectives and design of the study. The participants signed a written consent form for their participation. 

## RESULTS

A total of 96 participants took part in this study in three groups. The mean age of the mothers was 31.5±7.43 years and no significant differences were observed between the groups in terms of their demographic information (Table 1).

**Table 1:** Comparison of mothers in terms of demographic information in the three groups

	Group	Control group	Hospital tour	Oral instruction	P value	Test
Variables		N (%)	N (%)	N (%)		
Child Age (months)	12-20	8(25)	8(25)	9(28.1)	0.94*	KW=1.57
	21-29	11(34.40)	11(34.40)	11(34.40)		
	30-36	13(40.60)	13(40.60)	12(37.50)		
Child Sex	Girl	17(53.10)	16(50)	18(56.20)	0.88**	X^2^ =0.25
	boy	15(46.90)	16(50)	14(53.80)		Df=2
Child Disease	ASD^a^5(15.60)	3(9.40)	7(21.86)	0.81***	X2 =5.24
	VSD^b^8(25)	6(18.75)	6(18.75)			Df=2
	TOF^c^9(28.10)	8(25)	6(18.75)		
	PA^d^3(9.40)	6(18.75)	6(18.75)		
	PS^e^4(12.50)	3(9.40)	4(12.50)		
	AS^f^3(9.40)	5(15.60)	1(3.13)		
	MAPCA^g^0(0.0)	1(3.10)	1(3.13)		
	CMP^h^0(0.0)	0(0.0)	1(3.13)	
Length of Hospitalization (Days)	Less than 6	10(31.30)	11(34.40)	15(46.90)	0.41*	KW=1.77
	6-10	21(65.60)	18(56.2)	15(46.90)		
	More than 10	1(3.10)	3(9.40)	2(6.2)		
Mother’s age	20-30	16(50)	12(37.50)	15(46.90)	0.46*	KW=0.13
	31-40	15(46.90)	16(50)	12(37.50)		
	41-50	1(3.10)	4(12.50)	5(15.60)		
Mother’s education	Elementary School	2(6.2)	5(15.60)	6(18.75)	0.36*	KW=2.1
	Diploma	19(59.40)	17(53.10)	19(59.40)		
	Bachelor	11(34.40)	8(25)	6(18.75)		
	Masters	0(0.0)	2(6.30)	1(3.10)		
Number of children	1	10(31.30)	11(34.40)	14(43.75)	0.74*	KW=0.74
	2	16(50)	13(40.60)	11(34.40)		
	3	5(15.60)	6(18.75)	6(18.75)		
	4 or more	1(3.10)	2(6.25)	1(3.10)		
The history of hospitalization of child	yes	24(75)	26(81.25)	18(56.25)	0.07**	Df=2
	No	8(25)	6(18.75)	14(43.75)		X^2^=5.24

* Kruskal wallis test

** Chi-Square test

*** Fisher’s exact test

a ASD (Atrial Septal Defect)

b VAD(Ventricular Septal defect)

c TOF( Tetralogy of Fallot)

d PA(Pulmonary atresia)

e PS( Pulmonary stenosis)

f AS ( Aortic stenosis)

g MAPCA( Major Aortopulmonary Collateral Arteries)

h PDA( Patent Ductus Artriosus)

The Kolmogorov-Smirnov tests were used to assess the normality of anxiety scores. The results showed that the scores were normally distributed (P>0.05). The results showed no significant differences among the three groups before the intervention in terms of the mothers’ surgery-related (P=0.05) and state (P=0.18) anxiety scores. Based on the paired t-test, surgery-related anxiety reduced significantly in both the oral instruction and hospital tour groups after the intervention (P<0.001), while it increased in the control group, but non-significantly (P=0.92). The paired t-test also showed that the mothers’ state anxiety reduced significantly in both the oral instruction and hospital tour groups after the intervention (P=0.001), while it increased (P=0.41) in the control group. The ANOVA showed significant differences among the three groups after the intervention in terms of both surgery-related (P<0.001) and state (P<0.001) anxiety (Table 2). 

**Table 2:** Comparison of the mean surgery-related and state anxiety of mothers in the three groups before (24 hours before the operation) and after (one hour before operation) the intervention

	Group	Oral instruction	Hospital tour	Control group	P value*
Anxiety		Mean±SD	Mean±SD	Mean±SD	Between±SD
Operation-related	Before	14.25±3.95	13.87±2.36	15.68±2.58	0.05
	After	11.84±3.37	11.71±2.11	16.59±1.93	<0.001
P value** Within		<0.001	<0.001	0.92	
State	Before	46.75±4.50	48.09±4.91	48.84±4.22	0.18
	After	43.50±3.32	46.75±3.12	49.53±3.80	0.001
P value** Within		0.001	0.001	0.41	

* ANOVA

** Paired t test

The ANOVA also showed significant differences among the three groups in terms of the mean difference in the scores of surgery-related (P<0.001) and state (P<0.001) anxiety before and after the intervention (Table 3). 

**Table 3:** Comparison of mean difference of the scores of surgery-related and state anxiety of mothers in the three groups

Group	Oral instruction	Hospital tour	Control group	P value*	Test
	Mean±SD	Mean±SD	Mean±SD		
Mean difference of Surgery-related anxiety score, before and after intervention	2.4±1.74	2.1±2.01	-0.9±1.85	<0.001	F=30.99
Mean difference of State anxiety score, before and after intervention	3.2±4.7	1.2±4.15	0.6±4.66	<0.001	F=6.02

* ANOVA

According to the results of Tukey’s test, no significant differences were observed between the oral instruction and hospital tour groups (P=0.58) regarding the surgery-related anxiety scores. A significant difference was observed between the oral instruction and control groups (P<0.001) and also between the hospital tour and control groups (P<0.001) regarding the surgery-related anxiety scores. Based on the results of Tukey’s test, a significant difference was observed between the oral instruction and control groups (P=0.002) regarding the mothers’ state anxiety scores. There were no significant differences between the hospital tour and oral instruction groups (P=0.22) or between the hospital tour and control groups (P=0.18) regarding the mothers’ state anxiety scores.

## DISCUSSION

This study was conducted to evaluate the effect of taking a hospital tour on the anxiety of mothers before the open-heart surgery of their children. The results revealed that although the mean scores of surgery-related and state anxiety decreased significantly in both the oral instruction and hospital tour groups after the intervention, taking a hospital tour did not prove to be significantly different from receiving oral instructions. Compared to the conventional preparation group, however, both preparation methods reduced the mothers’ preoperative anxiety more effectively. 

The results revealed that although the mean scores of surgery-related and state anxiety decreased significantly in both the oral instruction and hospital tour groups after the intervention, taking a hospital tour did not show to be significantly different from receiving oral instructions. Compared to the conventional preparation group, however, both preparation methods reduced the mothers’ preoperative anxiety more effectively. 

Studies conducted on the effect of oral instructions confirm the results of this study. ^33 , 34^ Evidence indicates that holding preoperative instructional courses can result in reduced anxiety and improved compatibility. The results of a study conducted in Italy revealed that offering preoperative information in the form of oral instructions and demonstrating images of ICU appliances reduces the parents’ anxiety significantly compared to offering routine ward preparation. ^35^ The orderly and prescheduled structure of information presentation in oral instruction sessions appears to promote the learner’s knowledge and skills and exert positive effects on anxiety reduction. A previous three-group study proved that oral instructions using writing tools and images can play an important role in the reduction of preoperative anxiety in the parents. ^36^ Inconsistent with the findings of this study, in a quasi-experimental study of 300 randomly selected mothers, results showed that providing information in an oral form can reduce children’s concerns before general surgeries although it is not effective in reducing the parents’ preoperative anxiety. ^37^ This inconsistency in results could be attributed to the type of pediatric surgery taking place, the different tools used for the classes (video games and pamphlets, etc.), and the intervention performed on the operation day (rather than the day before).

The results of this study showed that taking a hospital tour is more effective than the conventional preparation method in reducing preoperative anxiety in the parents of children undergoing open-heart surgery. Studies have provided controversial results about the effectiveness of hospital tours on the patients and their family members’ anxiety. Some studies have reported positive effects for both virtual and real hospital tours on reducing the patients’ anxiety. ^19 , 24 , 38^ Another study that confirmed our results examined the effects of orientation tour (visiting the ICU and surgical unit) on state anxiety in candidates for coronary artery bypass and reported that the preoperative anxiety level had decreased. ^39^ Similarly, a study showed that in elective day surgery, visiting operating room with one parent was more effective than using the routine method in reducing the child’s anxiety. ^13^ In the above study, the tour was limited to visiting the operating room without providing any information, and the anxiety of children was investigated immediately before intravenous induction of anesthesia.

A previous study, however, demonstrated that a virtual tour rather increases the parents’ emotional distress and is not effective in reducing the children’s emotional distress either and is only beneficial in increasing the children’s awareness. ^23^ This inconsistency may be related to the differences in the type of the tour. Virtual tours provide information with more graphical and colorful details that may increase the parents’ level of emotional distress. Difference in the type of participants is also another probable reason for discordant results.

Likewise, another study showed that special educational sessions and a real tour of the cardiac surgery unit was not effective in reducing the patient’s preoperative anxiety. ^22^ The reason for this inconsistency can probably be explained by the difference in the method of intervention. In the above-mentioned study, immediately after the educational session, the patients participated in a tour, so they might not have been ready to take the tour. Another reason could be related to the timing of the tour. Immediate preoperative period may not have been suitable for preparation. ^33^

Regarding preoperative state anxiety in the mothers of children who have undergone open-heart surgery, the results of this study showed that oral instructions are significantly more effective than conventional preparation methods in reducing preoperative state anxiety, while hospital tours are not significantly different from the conventional preparation method. The results of a study on the effect of verbal and written preparation program on state anxiety in the parents of children undergoing surgery in a pediatric surgery outpatient clinic support the present findings. ^40^ Another two-group study showed that taking an ICU tour was not as effective as oral instructions on the anxiety of patients who had undergone heart surgeries and their family members; this study used the same assessment tools as the present study but did not show any significant reductions. ^41^ A recent clinical trial study conducted to compare two preparation methods on parents before their child’s dental surgery found no significant differences in parental anxiety between audiovisual method (animated video) and oral instructions groups. ^42^ However, the results showed that using oral instruction was as effective as audiovisual method on reducing preoperative parental anxiety. 

Although the different effect obtained for hospital tours on operation-related anxiety and state anxiety is a rather surprising finding of the present study, which requires further investigations, this different effect may be attributed to the nature of state anxiety. The reason is that state anxiety depends on the situation in question, as different situations lead to different degrees of tension and conflict and could be associated with a loss of self-control and a greater experience of anxiety and tension. ^30^ Surgery-related anxiety reduced more prominently than state anxiety in the hospital tour group in the present study perhaps because the subjects experienced the stressful environment of the operation room and observed the unfamiliar equipment and atmosphere of the operation room and ICU during the actual tour they were given, and these experiences might have been responsible for the reduced effectiveness of hospital tours on state anxiety. 

The limitations of the present study, which restricted the discussion part of this article and the comparison of the results with other studies, include the self-reported nature of the questionnaires used and the small number of studies on preparation through a hospital tour, especially in Iran. Furthermore, the present findings cannot be generalized to all the mothers of children who are the candidates of surgery. Two valid scales were used to assess anxiety, which can be considered as the strength of this study. 

## CONCLUSION

Conveying information to the mothers of children who have undergone open-heart surgery by oral instructions and image demonstration can reduce preoperative surgery-related anxiety as well as state anxiety compared to conventional preparation methods. The results of this study suggest the greater efficiency of oral instructions versus hospital tours in reducing these mothers’ anxiety. An implication of this study is that giving oral instructions to mothers before their children’s open-heart surgery can have positive outcomes in terms of reducing their preoperative anxiety. By adding to the existing knowledge, present results show that hospital tours have a different effect on state anxiety and operation-related anxiety in the mothers of children who are surgery candidates. Researchers recommend that physiological indicators of anxiety will also be examined in future studies. Based on the results, further studies are needed to investigate long-term effects of preoperative oral education in different clinical settings. 
